# Prevalence and Parental Risk Factors for Speech Disability Associated with Cleft Palate in Chinese Children—A National Survey

**DOI:** 10.3390/ijerph13111168

**Published:** 2016-11-23

**Authors:** Chunfeng Yun, Zhenjie Wang, Ping He, Chao Guo, Gong Chen, Xiaoying Zheng

**Affiliations:** Institute of Population Research, Peking University, Beijing 100871, China; chunfengyun@pku.edu.cn (C.Y.); zhenjie.wang@pku.edu.cn (Z.W.); pkuheping@pku.edu.cn (P.H.); chaoguo@pku.edu.cn (C.G.); chengong@pku.edu.cn (G.C.)

**Keywords:** cleft palate, children, risk factor, China

## Abstract

Although the prevalence of oral clefts in China is among the highest worldwide, little is known about the prevalence of speech disability associated with cleft palate in Chinese children. The data for this study were collected from the Second China National Sample Survey on Disability, and identification of speech disability associated with cleft palate was based on consensus manuals. Logistic regression was used to estimate odds ratios (ORs) and 95% confidence intervals (CIs). A weighted number of 112,070 disabled children affected by cleft palate were identified, yielding a prevalence of 3.45 per 10,000 children (95% CI: 3.19–3.71). A history of speech disability in the mother (OR = 20.266, 95% CI 5.788–70.959, *p* < 0.0001), older paternal child-bearing age (OR = 1.061, 95% CI 1.017–1.108, *p* = 0.0065, per year increase in age), and lower parental education (maternal: OR = 3.424, 95% CI 1.082–10.837, *p* = 0.0363; paternal: OR = 2.923, 95% CI 1.245–6.866, *p* = 0.0138) were strongly associated with risk of speech disability associated with cleft palate in the offspring. Our results showed that maternal speech disability, older paternal child-bearing age, and lower levels of parental education were independent risk factors for speech disability associated with cleft palate for children in China. These findings may have important implications for health disparities and prevention.

## 1. Introduction

Cleft palate is the most severe and disabling type of oral cleft, affecting about 17 of every 10,000 live-born babies worldwide [1]. Cleft palate not only has a significant impact on language development [2] but also may have adverse effects on their schooling attainment [3], social role understanding [4], and their caregivers mental health [5]. There are two main types of cleft palate: cleft lip with cleft palate (CLP) and isolated cleft palate (CP) [1]. Both types of clefts could be remedied significantly via proper and timely treatment, such as speech therapy and pharyngoplasty [2,6]. However, the opportunity for getting these treatments may be influenced by many socioeconomic factors, such as parental education, parental occupational categories, family income, and housing [7,8]. 

Cleft palate derives from an embryopathy. In the first 10 weeks of embryogenesis, the primary and secondary palate anastomose with each other and fuse in the midline of the nasal septum. Partial or complete failure of these structure to fuse leads to cleft palate [1]. Genetic and epidemiological studies have identified many risk factors, including genetic and environmental factors, for cleft palate. Genome-wide meta-analyses have identified several risk loci for cleft palate, including 1p36, 2p21, 3p11.1, 8q21.3, 13q31.1, and 15q22 [9]. Epidemiological studies have also identified some parental behavioral and environmental risk factors for cleft palate, including maternal active smoking [10] or passive smoking [11], maternal overweight or obesity before or during pregnancy [12,13], lack of nutrition and medical care during pregnancy [14], professional exposure to certain chemical pollutants [15], and residential proximity to industries [16]. Other studies have shown that older age at childbirth was a risk factor for cleft palate [17,18]. 

Several reports have indicated different rates (11.1 to 32.7 per 10,000 children) of oral cleft prevalence among Chinese children (for details, see Table S1). Some studies have used data from a Chinese birth defect survey that focused on oral cleft incidence among the entire population of live births and stillbirths from 28 weeks of gestation to 7 days after birth. Other studies have used data from hospital reports that also reported oral cleft incidence among newborns. However, little is known about the prevalence of speech disability in children associated with cleft palate in China. Moreover, the effect of parental and social risk factors on the risk of speech disability associated with cleft palate remains unclear. In the current study, we estimated the prevalence of speech disability associated with cleft palate among Chinese children aged 0–17 years and explored the associations between parental factors and the risk of cleft palate-related speech disability using a national population sample.

## 2. Methods

### 2.1. Study Population

The present sample was derived from a subpopulation of participants with cleft palate-related speech disability from the Second China National Sample Survey on Disability conducted from 1 April to 31 May 2006. This survey was a nationally representative study that used a stratified, multiphase, and cluster probability sampling design in the selection of participants. Moreover, it involved the random selection of 2526,145 persons in 771,797 households from 734 counties (cities or districts), 2980 towns (townships or streets), and 5964 communities that were selected from 31 provinces, autonomous regions, and municipalities directly under the Central Government in China (except Taiwan, Hong Kong, and Macau). All participants signed informed consent documents provided by Chinese State Council (Guo Ban Fa No 73 (2004)) [19].

### 2.2. Interviewers and Interviewing Procedures

More than 6000 doctors, more than 20,000 interviewers, and 50,000 survey assistants participated in this survey. Prior to the survey (before 25 March 2006), information about the number of households, population, suspected disabled people, and children aged 0–6 years in the sampling community was collected. 

To conduct the household survey, trained interviewers went to all selected households and interviewed every family member of the household. For subjects aged 7 years or older, a screen scale of disabilities was conducted by trained interviewers, and those suspected of having speech disability were then examined by neurologists following diagnostic manuals to confirm a final diagnosis, to assess the severity of the disability, if any, and to confirm its original causes. Children aged 0–6 years were first assessed by doctors in various specialties, and those suspected of having speech impairments were then examined by neurologists to confirm a final diagnosis of disability as well as the causes of disability. All children in the study were assessed by two specialized persons. The children aged 0–6 years were assessed by two doctors. Moreover, children aged 7–17 years were assessed by a trained interviewer and a doctor. An appointment was made for a second visit if any children were not at home during the interview. If the child was again unavailable on the subsequent visit, information on that person was obtained from the other family members.

### 2.3. Identification of Children with Speech Disability Associated with Cleft Palate

Cleft palate was diagnosed by the neurologist according to the manual established by medical experts. In agreement with this medical manual, the term *cleft palate* was used to refer to isolated cleft palate (CP), cleft lip with cleft palate (CLP), or cleft palate with anomalies of other systems. We excluded cases with cleft lip (CL) only. The term *speech disability* was used to refer to any type of language disorder present for children over 2 years of age that prevented them from taking part in normal language exchange and that negatively impacted their daily life, participation in social activities, or both [19].

### 2.4. Data Analysis

Standard weighting procedures were used to present population-weighted numbers, prevalence, and odds ratios (ORs) of speech disability associated with cleft palate among Chinese children [20]. Variance and corresponding 95% confidence intervals (CIs) were estimated by the Taylor series linearization method [21]. The chi-square test was used to compare prevalence between different groups.

Multivariable logistic regression models were used to calculate the adjusted ORs and 95% CIs. We considered both maternal and paternal factors on the risk of cleft palate for the offspring and included the following confounders: child’s gender, residence of the child (rural vs. urban), maternal and paternal education level, maternal and paternal age at conception of child (a continuous, untransformed variable), and history of speech disability in the mother and father. We used the procedures SURVEYFREQ and SURVEYLOGISTIC in the program SAS 9.1 to analyze data [22]. A *p*-value < 0.05 was considered statistically significant.

## 3. Results

### 3.1. Prevalence of Speech Disability Associated with Cleft Palate among Chinese Children

A total of 616,940 children aged 0–17 years were investigated in this survey. Above all, there were 9852 children with disability of any type identified. Moreover, there were 732 children with speech disability in this survey. The prevalence of speech disability among children aged 0–3, 4–6, and 7–17 years were 12.51, 25.21, and 9.11 per 10,000 children, respectively. Finally, the number of children with speech disability associated with cleft palate was 215. By the standard weighting procedures, the population-weighted number of children with speech disability associated with cleft palate was 112,070, yielding a weighted prevalence of 3.45 per 10,000 people (95% CI = 3.19–3.71). The weighted prevalence of speech disability associated with cleft palate among children aged 0–3, 4–6, and 7–17 years were 2.12, 5.65, and 3.37 per 10,000 children, respectively. Children aged 4–6 had a significantly higher rate than children aged 0–3 and 7–17 (χ^2^ = 17.25, *p* = 0.0005). More boys were affected by cleft palate than were girls (3.93 vs. 2.88 per 10,000 children, respectively; χ^2^ = 4.94, *p* = 0.0328), and there was also a significant difference in the prevalence for children in rural and urban areas (3.93 vs. 2.06 per 10,000 children, χ^2^ = 12.00, *p* = 0.0014). Additional details are presented in Table 1.

### 3.2. Parental Factors Associated with Speech Disability Associated with Cleft Palate among Chinese Children

A logistic regression model was used to explore the effect of both mother and father related factors on the risk of speech disability associated with cleft palate. Moreover, the following factors were associated with speech disability associated with cleft palate in Chinese children: maternal education level (junior middle school or less: OR = 3.424, 95% CI = 1.082–10.837, *p* = 0.0363 relative to senior middle school or more), paternal education level (junior middle school or less: OR = 2.923, 95% CI = 1.245–6.866, *p* = 0.0138 relative to senior middle school or more), maternal history of speech disability (OR = 20.266, 95% CI = 5.788–70.959, *p* < 0.0001, relative to no maternal history), gender of the child (male OR = 1.646, 95% CI = 1.157–2.342, *p* = 0.0056 relative to female), and paternal age at conception (OR = 1.061, 95% CI = 1.017–1.108, *p* = 0.0065 per year) (Table 2).

## 4. Discussion

This study revealed that the prevalence of speech disability associated with cleft palate was 3.45 per 10,000 Chinese children aged 0–17 years old, much lower than previous estimates [23]. The difference in sampling may account for the differences in prevalence. In previous studies, data were collected from newborns, which included 10%–20% stillbirths, dead fetuses, or babies who died within seven days (they always have much higher rate on cleft palate than live births) [23,24,25,26,27]. In this study, all the participants were still alive when the survey was performed.

The prevalence of speech disability associated with cleft palate among Chinese children aged 0–3 years was much lower than that in children aged 4–6 and 7–17 years. Because this survey was conducted in 2006, all the children aged 0–3 years had been born after 2002. Since 2002, many public welfare programs and organizations, such as the China Population Welfare Foundation, the Alliance for Smile, and the Smile Angel Foundation, have been active in the treatment of cleft palate in China. This may be one reason for the low prevalence of cleft palate-related speech disability among the 0–3 years cohort in our study. Another reason may be that it is difficult to diagnose speech disability in children younger than 3 years. In our study, the different interviewing procedures between children aged 0–6 and 7–17 years could have implications for the ascertainment of cases of speech disability associated with cleft palate. This may be another explanation for the differences in the prevalence by age group in the results.

In this study, we observed that cleft palate-related speech disability was more common in male than in female children. This significant difference in gender distribution is consistent with previous studies [23]. The cause of this gender difference in the prevalence of speech disability associated with cleft palate is unknown. One potential explanation is that craniofacial development varies according to gender; the palatal shelves in males are separated and vertical for a relatively shorter time than in females [28]. 

An interesting finding from our survey is the strong association between advanced paternal age at conception of children and the increased risk of cleft palate-related speech disability. Interestingly, our data suggest that only advanced paternal age is associated with cleft palate, whereas no significant relationship appeared between advanced maternal age at conception and the risk of cleft palate. It is now well established that male reproductive function declines with age [29,30]. Furthermore, a landmark study [31] published in 2012 identified that the diversity in the mutation rate of single nucleotide polymorphism (SNP) in the offspring increased by two mutations per year based on paternal age at conception of children. This study also provides compelling evidence that fathers may transmit a much higher number of mutations to their offspring than do mothers [31]. During the aging process, male germ cells appear to change in both number and at a molecular level [29]. Furthermore, most of the known risk loci for cleft palate are located in the euchromosome that consists in the male germ cells [9,32,33], particularly for the Chinese population [34,35]. Therefore, mutations in male germ cells may be an explanation for the fact that advanced paternal age was associated with an increased risk of cleft palate. In addition, our findings were similar to former analyses from surveys that included large samples of live births [17,36], which also found that advanced paternal age, but not advanced maternal age, was associated with the risk of cleft palate. In a meta-analysis, Herkrath et al. found similar results: fathers 40 years of age or older had a 58% higher probability of having a child with cleft palate compared to fathers aged between 20 and 39 years, whereas mothers aged 40 years or over were only 1.56 times more likely to have a newborn with cleft lip with or without cleft palate compared with those aged between 20 and 29 years [18].

A family history of speech disability in parents was found to be strongly associated with cleft palate-related speech disability in children. As mentioned earlier, the cleft palate is a disease of polygenic inheritance [37]. This may be a major explanation for the association with family history. These findings were similar to prior results [38]. We noted that parents with speech impairment posed a greater risk for children with cleft palate-related speech disabilities. However, in the combined model that included both paternal and maternal factors, a history of speech disability in the father became insignificant. Thus, speech disability in the mother may be a much important risk factor. 

Our research indicated that both low maternal and paternal education levels appeared to be risk factors for offspring with speech disability associated with cleft palate. This finding was similar to some former studies that showed that low parental education increased the risk of oral clefts in the offspring [38,39]. Good health depends on recognizing the risks of various lifestyles, and more education leads to a healthier lifestyle by means of creative work and a sense of controlling one’s own life [40]. A person with less education has a greater likelihood of being exposed to secondhand smoke [41] or working in hostile conditions [42]. Unhealthy lifestyle elements such as these are not good for offspring [43] and may contribute to congenital defects such as cleft palate [15,44]. Furthermore, parental education level is one measure of neighborhood-based socio-economic status (SES), which was associated with the risk of oral cleft [8,45].

This study is limited by the fact that all children in the selected households were first screened for any kinds of disability, and only those suspected of having speech disability were examined by the neurologists to confirm whether the speech disability was associated with cleft palate. Therefore, children with cleft palate who were not disabled might not have been identified during the survey, which would result in an underestimation of the prevalence of cleft palate. To the best of our knowledge, previous studies on the prevalence of cleft palate in Mainland China were all based on hospital samples, which may introduce referral bias. However, this study was based on a national survey with wide geographic coverage and sufficient sample size, and this analysis should provide an accurate prevalence of speech disability associated with cleft palate among Chinese children. However, because of the lack of relevant information on prenatal or perinatal factors (e.g., smoking, drinking, prenatal diagnosis, and medical care), this study failed to explore any associations between cleft palate and these factors in Chinese children. As the questionnaire did not differentiate types of cleft palate, the detail number of children with cleft palate and cleft lip and palate remains unknown. This may be another limitation of this study.

## 5. Conclusions

Our study is the first to report on cleft palate-related speech disability among Chinese children. The prevalence was 3.45 per 10,000 among Chinese children aged 0–17 years. Independent risk factors include maternal speech disability, advanced paternal age at conception, and lower levels of maternal and paternal education. Greater public attention to the oral health of offspring is required among those with less education. Older fathers should pay greater attention to the risk of cleft palate in their offspring.

## Figures and Tables

**Table 1:** Prevalence of speech disability associated with cleft palate in Chinese children.

	Weighted Number (*n*)	Weighted Prevalence (per 10,000 People, 95% CI)	*p*-Value
Age group			0.0005
0–3	12,335	2.13 (1.66–2.60)	
4–6	24,526	5.65 (4.77–6.53)	
7–17	75,209	3.37 (3.07–3.67)	
Gender			0.0328
Male	68,709	3.94 (3.57–4.31)	
Female	43,361	2.88 (2.55–3.21)	
Residence location			0.0014
Rural	94,909	3.93 (3.61–4.25)	
Urban	17,161	2.06 (1.67–2.45)	
Total	112,070	3.45 (3.19–3.71)	

CI: Confidence Interval.

**Table 2:** Parental factors associated with cleft palate caused disability in Chinese children.

Variable	Odds Ratio (95% CI)	*p*-Value
Children Gender		
Male	1.646 (1.157–2.342)	0.0056
Female	Ref.	-
Residence location		
Rural area	1.159 (0.715–1.881)	0.5494
Urban area	Ref.	-
History of speech disability in mother		
Yes	20.266 (5.788–70.959)	<0.0001
No	Ref.	-
History of speech disability in father		
Yes	5.334 (0.637–44.656)	0.1226
No	Ref.	-
Maternal education level		
Junior middle school or under	3.424 (1.082–10.837)	0.0363
Senior middle school or above	Ref.	-
Paternal education level		
Junior middle school or under	2.923 (1.245–6.866)	0.0138
Senior middle school or above	Ref.	-
Maternal age at conception of child (per year)	0.970 (0.924–1.018)	0.2124
Paternal age at conception of child (per year)	1.061 (1.017–1.108)	0.0065

Adjusted for children’s single age. Ref.: reference category.

## Supplementary Materials

The following are available online at www.mdpi.com/1660-4601/13/11/1168/s1. Table S1: Studies on the prevalence of oral cleft in China.
